# Evaluation of the immunological profile of antibody-functionalized metal-filled single-walled carbon nanocapsules for targeted radiotherapy

**DOI:** 10.1038/srep42605

**Published:** 2017-02-15

**Authors:** Aritz Perez Ruiz de Garibay, Cinzia Spinato, Rebecca Klippstein, Maxime Bourgognon, Markus Martincic, Elzbieta Pach, Belén Ballesteros, Cécilia Ménard-Moyon, Khuloud T. Al-Jamal, Gerard Tobias, Alberto Bianco

**Affiliations:** 1 University of Strasbourg, CNRS, Immunopathology and Therapeutic Chemistry, UPR 3572, 67000 Strasbourg, France; 2 Institute of Pharmaceutical Science, Faculty of Life Sciences & Medicine, King’s College London, London SE1 9NH, UK; 3 Institut de Ciència de Materials de Barcelona (ICMAB-CSIC), Campus UAB, 08193, Bellaterra, Barcelona, Spain; 4 Catalan Institute of Nanoscience and Nanotechnology (ICN2), CSIC and The Barcelona Institute of Science and Technology, Campus UAB, Bellaterra, 08193 Barcelona, Spain

## Abstract

This study investigates the immune responses induced by metal-filled single-walled carbon nanotubes (SWCNT) under *in vitro, ex vivo* and *in vivo* settings. Either empty amino-functionalized CNTs [SWCNT-NH_2_ (**1**)] or samarium chloride-filled amino-functionalized CNTs with [SmCl_3_@SWCNT-mAb (**3**)] or without [SmCl_3_@SWCNT-NH_2_ (**2**)] Cetuximab functionalization were tested. Conjugates were added to RAW 264.7 or PBMC cells in a range of 1 μg/ml to 100 μg/ml for 24 h. Cell viability and IL-6/TNFα production were determined by flow cytometry and ELISA. Additionally, the effect of SWCNTs on the number of T lymphocytes, B lymphocytes and monocytes within the PBMC subpopulations was evaluated by immunostaining and flow cytometry. The effect on monocyte number in living mice was assessed after tail vein injection (150 μg of each conjugate per mouse) at 1, 7 and 13 days post-injection. Overall, our study showed that all the conjugates had no significant effect on cell viability of RAW 264.7 but conjugates **1** and **3** led to a slight increase in IL-6/TNFα. All the conjugates resulted in significant reduction in monocyte/macrophage cell numbers within PBMCs in a dose-dependent manner. Interestingly, monocyte depletion was not observed *in vivo*, suggesting their suitability for future testing in the field of targeted radiotherapy in mice.

In the past years, taking advantage of their unique physicochemical properties, both multi-walled carbon nanotubes (MWCNTs) and single-walled carbon nanotubes (SWCNTs) have emerged as promising tools for biomedical applications^1^,^2^,^3^,^4^. For instance, their favorable electrical properties and their responsiveness to changes in the surrounding environment allow carbon nanotubes (CNTs) to act as promising biosensors^5^. In addition, thanks to their small diameter, high aspect ratio and toughness, CNTs have been proposed as atomic force microscopy (AFM) tip nanoinjectors to facilitate the insertion of molecules into cells in a target point^6^,^7^. Moreover, due to their capability to penetrate into cell membranes^8^,^9^, CNTs can work as efficient gene delivery systems^10^,^11^,^12^,^13^,^14^,^15^. They have been reported to promote gene silencing by forming supramolecular complexes between cationic functionalized CNTs and short RNA oligomers, which could help in the treatment of cancer or immune diseases^10^,^11^,^12^,^13^,^14^,^15^. In a similar way, with the appropriate functionalization, CNTs can deliver drugs to specific target cells (*i.e*. cancer cells) in order to enhance the effect of the drug they are carrying as it was demonstrated for several CNT-doxorubicin complexes^16^,^17^,^18^. Comparing the number of studies conducted in those fields, little work has been carried out regarding the use of CNTs as vectors for radionuclides to be exploited either for cancer diagnosis or therapy^19^,^20^,^21^,^22^,^23^,^24^,^25^. Moreover, all these studies have focused on the attachment of chosen moieties to the external walls, but we can take also advantage of the presence of the inner cavity to host biomedically relevant payloads^26^. In fact, it is possible to fill the nanotubes with a therapeutic or imaging cargo whilst the external wall remains available for their derivatization with dispersing and targeting molecules. Encapsulation of molecules inside the nanotubes also has the advantage of protecting and isolating them from the external environment and avoid their free circulation in the body. Among the many examples of filled CNTs for bioapplications, most researches have focused on the encapsulation of drug molecules for therapeutic purposes, while only few have described biomedical imaging with magnetic nanoparticles (for magnetic resonance imaging)^27^ and radionuclides (*via* single-photon emission computed tomography)^28^.

We have recently reported a complete study describing the design of antibody-functionalized SWCNTs filled with radioactivable metals towards targeted anticancer therapy^29^. To this purpose, steam-purified SWCNTs were filled with samarium or lutetium chloride. After high-temperature sealing, SWCNTs were covalently functionalized with the monoclonal antibody (mAb) Cetuximab (Erbitux^®^) targeting the epidermal growth factor receptor (EGFR), overexpressed on several cancer cells. Our study highlighted the great possibilities offered by these filled and functionalized CNTs, which were able to internalize more efficiently into EGFR positive cancer cells. Moreover, these findings prompted us to lead a thorough investigation of the immunological impact of these conjugates.

A suitable nanomaterial should prevent several outcomes in order to be biocompatible, such as triggering immune reactions, acute inflammatory responses or cytotoxicity within the cells to which it is targeted, or cells of first-line exposure^30^. Classically, two lines of defense are known. Innate immunity is the first activated line responsible for combating foreign organisms or substances, mainly *via* complement activation and macrophage and neutrophil actions. This leads to the elimination of the intruders and further activation of the adaptive immunity. Adaptive responses, on the other hand, are durable specific reactions triggered by T and B lymphocytes. It is crucial to study the impact of nanomaterials, including CNTs, on these immune cells and the outcomes of this encounter^31^. A recent review has reported a helpful overview on the immune impact of carbon nanomaterials to guide future research on their immunological applications in biomedicine^32^. The studies of the effects on the immune systems are not limited only to carbon materials. Other types of nanoparticles and nanomaterials may result immune compatible or could exert an immune specific action depending on their surface functionalization and chemical composition^33^,^34^,^35^.

This work aimed to analyze the immunological profiles of a wide range of doses of three different SWCNT conjugates: empty amino-functionalized CNTs [SWCNT-NH_2_ (**1**)] and samarium chloride-filled amino-functionalized CNTs without [SmCl_3_@SWCNT-NH_2_ (**2**)] or with [SmCl_3_@SWCNT-mAb (**3**)] Cetuximab functionalization in murine (RAW 264.7 macrophages) and human cells (peripheral blood mononuclear cells, PBMCs). Parameters tested *in vitro* include viability, cell activation and cytokine production. Additionally, the effects of the conjugates on PBMC viability and number of cell subpopulations [T lymphocytes (L_T_), B lymphocytes (L_B_) and monocytes/macrophages] were evaluated. Finally, the percentage of monocyte/macrophage population within PBMCs after tail vein injection of the conjugates in C57Bl/6 mice was determined.

## Results

### Functionalization of SWCNTs

**Figure 1:**
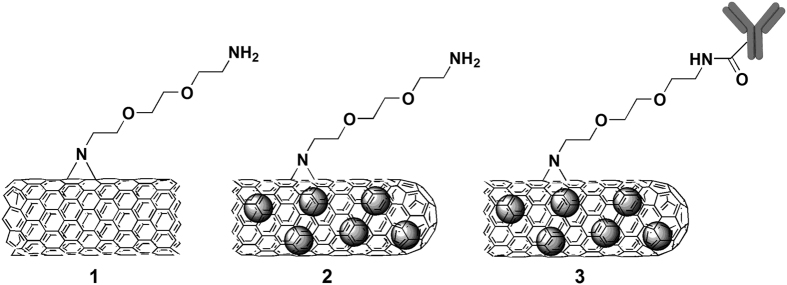
Molecular structures of SWCNT-NH_2_ (1), SmCl_3_@SWCNT-NH_2_ (2) and SmCl_3_@SWCNT-mAb (3). The left-side open end of compounds **2** and **3** is meant to visually indicate the extended length of the nanotubes.

**Figure 2:**
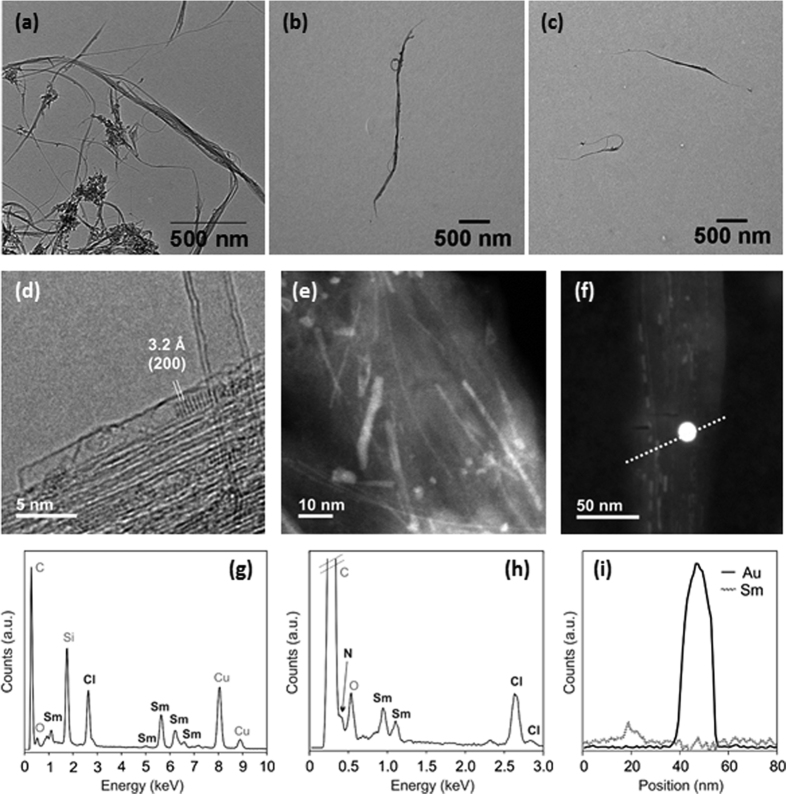
Electron microscopy characterization of empty and SmCl_3_ filled SWCNT conjugates. Low resolution TEM images of pristine SWCNTs (**a**), Pht-protected SWCNT-NH_2_ **1** (**b**) and SWCNT-NH_2_ **1** (**c**). (**d**,**g**) HRTEM image of SmCl_3_@SWCNT precursor and corresponding EDX spectrum. (**e**,**h**) HAADF-STEM image of SmCl_3_@SWCNT-NH_2_ (**2**) showing the bright lines arising from the filling material and EDX spectrum showing the nitrogen signal associated with the attached amino functional groups. (**f**,**i**) Sm and Au EDX line profiles acquired in STEM mode showing the location of the filling compound and the gold nanoparticles (AuNPs) in SmCl_3_@SWCNT-mAb (**3**) after being immunostained with a secondary antibody conjugated with AuNPs.

In this study we have compared the immunological impact of three different types of functionalized SWCNTs: SWCNT-NH_2_ (**1**), SmCl_3_@SWCNT-NH_2_ (**2**) and SmCl_3_@SWCNT-mAb (**3**). To evaluate the eventual effect of the presence of filling material (SmCl_3_) on cells, we have employed both empty and samarium-filled CNTs. Purified and shortened SWCNTs were initially functionalized by nitrene cycloaddition with amino-terminating triethylene glycol (TEG) chains, with the aim of increasing their water dispersibility and biocompatibility^29^. The free amine loading of SWCNT-NH_2_ (**1**) and SmCl_3_@SWCNT-NH_2_ (**2**) calculated by the Kaiser test was 104 μmol/g and 90 μmol/g, respectively. Functionalized SmCl_3_-filled CNTs **2** were then further derivatized with the targeting antibody Cetuximab by coupling reaction on the TEG terminal amino group, obtaining SmCl_3_@SWCNT-mAb (**3**). The structural representation of the three conjugates is shown in Fig. 1. The morphological characterization of all conjugates was carried out using different spectroscopic and microscopic techniques. TEM images of the precursors of empty SWCNT-NH_2_ (**1**) confirmed that the nanotubes remained structurally intact throughout all functionalization steps (Fig. 2). The comparison of the thermogravimetric analysis between the precursor pristine nanotubes and the protected SWCNT-NH_2_ (**1**) allowed to assess the degree of functionalization (Figure S1). The filled SmCl_3_@SWCNT-NH_2_ (**2**) and SmCl_3_@SWCNT-mAb (**3**) correspond to the same batches of tubes reported in our previous work^29^. Additional characterizations using high resolution transmission microscopy (HRTEM), high-angle annular dark-field scanning transmission electron microscopy (HAADF-STEM) and energy-dispersive X-ray spectroscopy (EDX) are shown in Fig. 2. HRTEM images of SmCl_3_@SWCNT precursors (Fig. 2d) confirmed that the encapsulated metal halide is crystalline and its structure is in good agreement with the hexagonal P6_3_/m structure of the bulk material. After functionalization with amine groups the SWCNTs remained filled as shown in the HAADF-STEM image of sample SmCl_3_@SWCNT-NH_2_ (**2**) (Fig. 2e). Intensity in HAADF-STEM images is proportional to the atomic number, therefore the metal halide, heavier than the carbon from the SWCNTs, appears as bright lines. The presence of the amine groups in this sample is proven by EDX spectroscopy, where the signal corresponding to nitrogen is observed as a shoulder at 0.4 keV (Fig. 2h). To assess the functionalization with the antibody Cetuximab, SmCl_3_@SWCNT-mAb (**3**) was immunostained with a secondary antibody conjugated with gold nanoparticles (AuNPs)^29^. Both the AuNPs and the samarium chloride filling are visible with bright intensity in the HAADF-STEM image in Fig. 2f. EDX composition profiles demonstrate that the large bright dots correspond to the AuNPs, and their location on the SWCNTs is an indication of the successful attachment of the antibody Cetuximab onto the SmCl_3_ filled SWCNTs. Overall, these analyses confirm that the structural integrity was not affected during the organic and biological functionalization of the filled SWCNTs^29^. The three conjugates differ either in the absence or presence of the samarium chloride filling (conjugates **1** and **2**, respectively), the presence of open or closed ends (conjugates **1** and **2**, respectively)^36^ or the absence or presence of targeting antibody (conjugates **2** and **3**, respectively).

### Viability, cell activation and cytokine production in RAW 264.7 macrophages *in vitro*

**Figure 3:**
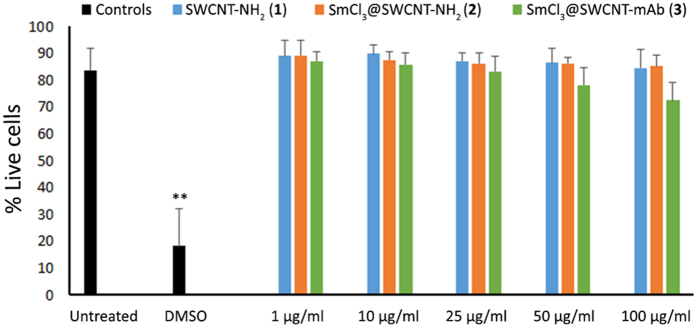
Viability of RAW 264.7 macrophages. Cells were incubated with the three SWCNT conjugates for 24 h at increasing concentrations (1, 10, 25, 50 and 100 μg/ml). DMSO (20%) was used as a positive control of death. Cell viability was determined with Annexin V/Propidium iodide staining and quantified by flow cytometry and no significant differences were observed for all compounds after 24 h of incubation (n = 3). Values are expressed as mean ± SD. **p < 0.01 with respect to untreated cells.

We previously reported that SmCl_3_@SWCNT-NH_2_ (**2**) and SmCl_3_@SWCNT-mAb (**3**) did not display cytotoxic effects in U87 and CHO cells^29^. However, a broader analysis on immune cells is needed to demonstrate the safe use of these conjugates for future applications in the biomedical field. For this purpose, the first step was to explore the impact of the SWCNT conjugates *in vitro* on the murine RAW 264.7 macrophage cell line. After 24 hour incubation with 1, 10, 25, 50 and 100 μg/ml of either of the conjugates, no significant reduction in overall cell viability (Fig. 3) or apoptotic cell numbers (Supplementary Figure S2) was observed.

**Figure 4:**
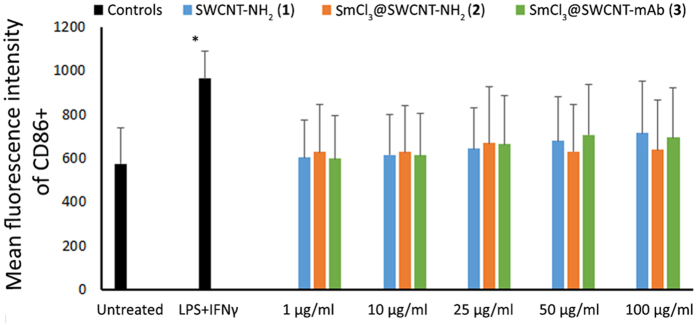
Activation of RAW 264.7 macrophages. RAW 264.7 cells were incubated with the SWCNT conjugates for 24 h at increasing concentrations (1, 10, 25, 50 and 100 μg/ml), then stained for CD86+ expression. LPS combined with IFN-γ was used as a positive control of activation. Mean fluorescence intensity of CD86+ cells was measured to express degree of cell activation, following treatments with the conjugates. No significant differences were observed for any of the treatments. Values are expressed as mean ± SD (n = 3). *p < 0.05 with respect to untreated cells.

Since one of the main roles of immune cells is to respond to foreign bodies, cell activation was investigated by analyzing expression of CD86 marker, a co-stimulatory molecule expressed in activated macrophages. SWCNT exposure to cells did not cause cellular activation with any of the conjugates at all concentrations tested (p > 0.05) (Fig. 4).

**Figure 5:**
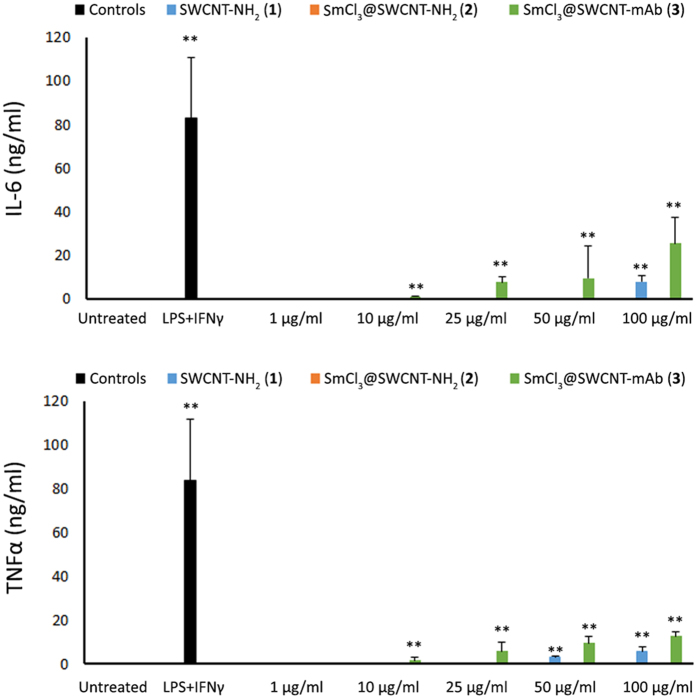
Cytokine production by RAW 264.7 macrophages. RAW 264.7 macrophages were incubated with the SWCNT conjugates for 24 h at increasing concentrations (1, 10, 25, 50 and 100 μg/ml), followed by measurements of IL-6 (top) and TNFα (bottom) cytokine production by ELISA. Values are expressed as mean ± SD (n = 3). The absence of bars indicates negligible levels. **p < 0.01 with respect to untreated cells.

Macrophage activation leads to production of pro-inflammatory cytokines. Titers of IL-6 and TNFα present in the supernatants of the treated cells, determined by ELISA, showed negligible values for both cytokines for cells treated with SmCl_3_@SWCNT-NH_2_ (**2**), similar to untreated cells (Fig. 5). Only a small but significant increase in both cytokines was detected in cells treated with SWCNT-NH_2_ (**1**) or SmCl_3_@SWCNT-mAb (**3**). These values however were at least 3-fold lower than values obtained in the positive control. Altogether, these results suggested low toxicity threshold for SWCNTs (**1**–**3**) in RAW 264.7 macrophages.

### Effect of SWCNTs on human PBMC subpopulations *ex vivo*

**Figure 6:**
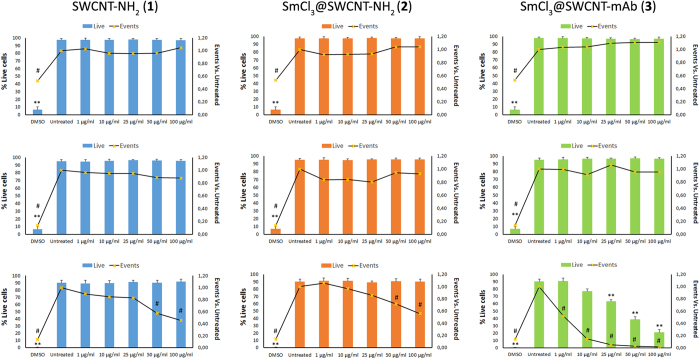
Effect of treatments with conjugates on PBMC viability and subpopulation cell numbers. PBMCs were incubated with the SWCNT conjugates for 24 h at increasing concentrations (1, 10, 25, 50 and 100 μg/ml), followed by flow cytometry measurements *via* propidium iodide staining to determine cell viability. Further staining was performed for CD3 (T lymphocytes, L_T_), CD19 (B lymphocytes, L_B_) or CD14 (monocytes/macrophages) to determine cell numbers within each of the subpopulation by flow cytometry. DMSO was used as a positive control. Percentage cell viability (bars) (PI staining) and number of positive events (black lines) are shown for L_T_ CD3+, L_B_ CD19+ and monocytes/macrophages CD14+ (from top to bottom). Values are expressed as mean ± SD (n = 3). **p < 0.01 with respect to untreated cells (% live cells). ^#^p < 0.05 with respect to untreated cells (events vs. untreated).

As previously suggested, the cytotoxic effects of SWCNTs depend on the cell type^37^. Therefore, we continued our study using human primary PBMCs, which were obtained by Ficoll-Histopaque density gradient centrifugation of leukocyte-rich buffy coats from healthy adult donors. PBMCs were cultured for 6 hours before being treated with 1, 10, 25, 50 and 100 μg/ml of either of the conjugates for 24 hours. Then, viability was evaluated by flow cytometry using Annexin V and propidium iodide. In addition, cytokine levels were also analyzed from culture supernatants. Taking into account that the 3 types of cells (*i.e*. L_T_, L_B_ and monocytes/macrophages) constituting the PBMCs are unlikely to react equally to SWCNTs, each of the subpopulations, tagged with cell specific fluorescent markers were analyzed separately by flow cytometry. None of the conjugates affected the overall cell viability or L_T_ and L_B_ population cell numbers (number of events) (Fig. 6). Exceptionally, the CD14+ population (monocytes/macrophages) was adversely affected. Cell counts were significantly reduced in a concentration-dependent manner starting at concentrations >50 μg/ml [SWCNT-NH_2_ (**1**) and SmCl_3_@SWCNT-NH_2_ (**2**)] and >1 μg/ml (SmCl_3_@SWCNT-mAb (**3**)]. The viability of cells treated with the latter was also affected at concentrations higher than 25 μg/ml.

**Figure 7:**
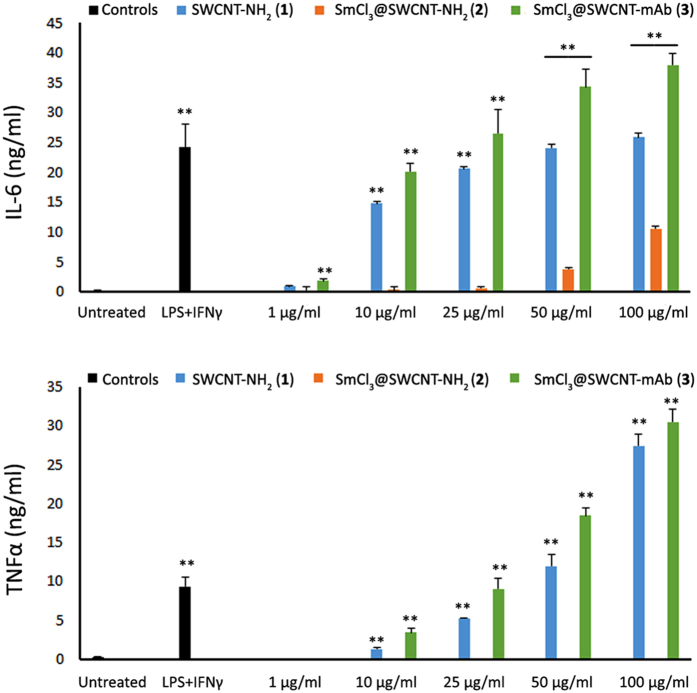
Cytokine production by PBMCs. PBMCs were incubated with the SWCNT conjugates for 24 h at increasing concentrations (1, 10, 25, 50 and 100 μg/ml), followed by measurements of IL-6 (top) and TNFα (bottom) cytokine production by ELISA. Values are expressed as mean ± SD (n = 3). The absence of bars indicates negligible levels. **p < 0.01 with respect to untreated cells.

These results were further confirmed upon cytokine analysis where prominent increase in IL-6 and TNFα production was observed, more significantly with SWCNT-NH_2_ (**1**) and SmCl_3_@SWCNT-mAb (**3**), in a concentration-dependent manner with comparable values to that of the positive control reaching the same levels or sometimes surpassing the positive control (LPS + IFN-γ) (Fig. 7).

### Immunological impact of SWCNTs after *in vivo* administration

A pilot study in C57Bl/6 mice was undertaken to examine if the *in vitro* findings of this study are translated *in vivo* but using more therapeutically relevant doses. Five groups of 4 mice were injected with either 150 μl of PBS (negative control), 3 mg/kg LPS (positive control) or 150 μl of a 1 mg/ml SWCNT dispersions, all *via* the tail vein except for the positive control.

The number of events of L_T_ (CD3+), L_B_ (CD45R/B220+) and monocytes/macrophages (CD11b+) in the PBMCs were analyzed at specific time points post-treatment (1, 7 and 13 days). In case of acute immunological reaction *in vivo*, an increase in the PBMC numbers is expected^21^.

**Figure 8:**
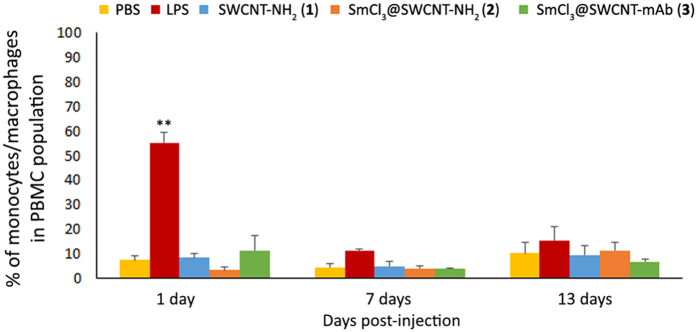
Percentage of monocyte/macrophage population within PBMCs after tail vein injection of the conjugates in mice. C57Bl6 mice were injected *via* the tail vein with 150 μl of one of the conjugates (1 mg/ml), PBS (negative control), LPS (3 mg/kg) (positive control). Whole blood was collected at 1, 7 and 13 days post-injection and cells were stained for CD3e (L_T_), CD45R/B220 (L_B_) or CD11b (monocytes/macrophages) expression. Percentage of CD11b+ cells (monocytes/macrophages) in PBMCs was calculated. Values are expressed as mean ± SD (n = 4). **p < 0.01 with respect to the other samples.

Similar to what was observed in human PBMCs, no changes in the L_T_ and L_B_ populations cell number at any of the time points tested were found (data not shown). Unlike the *ex vivo* studies using human PBMCs, the murine monocytes population was not affected (Fig. 8). The positive control group showed an increased number of monocytes at 24 hours in comparison to the PBS-treated mice. These values returned to baseline at 7 days onwards post-treatment. The negative immune response following SWCNT treatments agreed with the cytokine production profiles (Supplementary Figure S3). No changes in weight mice was observed (data not shown).

## Discussion

In the present study we have explored the immune response of three differently functionalized SWCNT conjugates at increasing concentrations in three experimental setups: *in vitro, ex vivo* and *in vivo*. For this purpose we have selected a sample of empty amino-functionalized CNTs, SWCNT-NH_2_ (**1**), a sample of samarium chloride-filled amino-functionalized CNTs, SmCl_3_@SWCNT-NH_2_ (**2**), and a sample of filled CNTs further functionalized with the targeting antibody Cetuximab, SmCl_3_@SWCNT-mAb (**3**). Conjugates **1** and **2** were chosen to investigate possible effects determined by the presence of the filling material, since they are both functionalized with amino-terminating functionalities. SmCl_3_@SWCNT-mAb (**3**), represents a third conjugate type, possessing a bioactive moiety, and was obtained by derivatization of compound **2**. A concentration range between 1 and 100 μg/ml was selected based on our previous findings^29^. The highest dose was supposed to trigger a certain degree of cytotoxicity and it was necessary to determine the range of concentration that can be subsequently selected in a biomedical context (i.e. few tens of μg/ml of injectable nanotubes)^1^.

Initial studies focused on analyzing cell viability, cell activation and production of cytokines using a murine macrophage cell line, following their incubation with SWCNTs *in vitro*. The assessment of macrophage response to CNTs is a central component in the evaluation of newly synthesized derivatives as it is known that these cells can quickly engulf CNTs, depending on their size and charge, contributing to the generation of an immune response. Following these initial studies, an *ex vivo* approach relying on human PBMCs from healthy donors was used. Cell viability and ability of CNTs to stimulate one or more of the cell subpopulations (cell numbers) were investigated. This was confirmed by measuring IL-6 and TNFα production triggered by exposure to SWCNTs. Finally, an *in vivo* pilot study was performed in C57Bl/6 mice to assess the impact of the conjugates on the PBMCs isolated at different time points.

Our results did not show remarkable effects on the viability of RAW 264.7 cells (Fig. 3), their activation (Fig. 4) or the production of cytokines (Fig. 5) even at high doses (up to 100 μg/ml) of CNTs. A variable effect on cytokine production was noticed, especially on the IL-6 production but none reached the titres of the positive control. While SmCl_3_@SWCNT-NH_2_ (**2**) had only a negligible effect on cytokine release, SWCNT-NH_2_ (**1**) and SmCl_3_@SWCNT-mAb (**3**) induced a small, but significant IL-6 production. The behavior of RAW 264.7 cells reported in the literature in response to SWCNTs is controversial. While some authors reported a cytotoxic effect with doses below 20 μg/ml^38^, other researchers did not find any evidence to support these outcomes^28^. Our study was in agreement with the *in vitro* observations by Shvedova *et al*. using this cell line. In their study, SWCNTs produced significantly lower levels of TNFα and IL-1β than the positive control and did not significantly trigger apoptosis^39^.

The minimal effects of SWCNT-NH_2_ (**1**) and SmCl_3_@SWCNT-mAb (**3**) appeared more pronounced when using human PBMCs, with monocyte/macrophage population showing the highest reduction in number (Fig. 6). The high titers of IL-6 and TNFα measured under these conditions are in agreement with these results (Fig. 7). A major effect was expected in this population for several reasons: (i) monocytes/macrophages are adherent cells and this might favor their interaction with CNTs; (ii) these types of phagocytic cells tend to internalize higher amounts of CNTs in comparison to LT and LB^40^,^41^,^42^; and (iii) CNTs have been shown to specifically activate monocytes, but not other immune cells (i.e. T lymphocytes), by analyzing their gene expression profiles^43^.

In the case of CNTs, different parameters can affect cell behavior, including the degree of dispersibility, the length of the CNTs, the amount of functional groups or the type of cells analyzed^37^. Besides, it is important to note that the toxic effects are normally dependent on the administered doses of CNTs^44^,^45^. As it has been previously reported, pristine, non-functionalized long CNTs are toxic^39^,^46^,^47^, while functionalization has been shown to decrease the toxic effect in several studies^42^,^48^,^49^,^50^,^51^. In fact, Dumortier *et al*. reported that functionalized SWCNTs do not alter the immune response of murine primary cells^42^. In another study, Delogu *et al*. demonstrated that different types of MWCNTs did not show cytotoxic effects on a wide variety of primary human immune cells^52^.

The fact that conjugates **1** and **3** exerted more pronounced immunological effect than conjugate **2** can be attributed to a couple of factors. SWCNT-NH_2_ (**1**) and SmCl_3_@SWCNT-NH_2_ (**2**) differ by two characteristics: the absence/presence of the filling and the open or closed ends, respectively. While the filling may not have, in this case, any harmful effect, the open-ended nature of SWCNT-NH_2_ (**1**) may have resulted in an increased cytotoxicity. This could be explained taking into account that the end caps of open SWCNTs may have several dangling bonds, which are highly reactive sites^53^ that tend to interact and affect cell integrity. Differently, in SmCl_3_@SWCNT-NH_2_ (**2**), the majority of the tubes are closed and devoid of dangling bonds, as a result of the employed molten phase filling process which was performed at high temperature (900 °C). In fact, during the cooling step which follows the high-temperature filling, the tips of the CNTs close on themselves engendering sealed tubes^36^. The close-ended filled nanotubes are also referred to as “carbon nanocapsules”. This might have resulted in SmCl_3_@SWCNT-NH_2_ (**2**) being more biocompatible. Conjugates (**1**) and (**2**) were obtained by functionalization of the corresponding steam-treated SWCNTs with a TEG precursor^29^. TEG chains on the sidewalls of the SWCNTs (Fig. 1) generally increase the water-dispersibility of the CNTs thanks to their hydrophilic nature. The preferred use of a short linker, TEG, over the long-chained polyethyleneglycol (PEG) in this instance was solely based on the ease of synthetic reaction using the former; unreacted PEG was found difficult to be removed from SWCNTs following chemical functionalization. Unexpectedly, these chains did not completely fulfill the requirements in terms of dispersibility. However, we considered the dispersibility of the conjugates to be sufficiently high to perform the different *in vitro* and *in vivo* experiments (Supplementary Figure S4).

SmCl_3_@SWCNT-NH_2_ (**2**) was further derivatized with the targeting antibody Cetuximab by a coupling reaction on the terminal amino group, yielding SmCl_3_@SWCNT-mAb (**3**). This functionalization step, intended to specifically target SmCl_3_@SWCNT-mAb (**3**) to EGFR-expressing cells as previously reported^29^, resulted in an improved dispersibility in physiological media. We hypothesize that the improved aqueous dispersibility of this conjugate compared to SWCNT-NH_2_ (**1**) and SmCl_3_@SWCNT-NH_2_ (**2**) resulted in a more efficient cell internalization of the former. This could have indirectly resulted in a significantly higher cytotoxic effect of (**3**) than (**1**) and (**2**), in the murine monocyte/macrophage population. Similar conclusions were previously obtained by other authors, where oxidized CNTs resulted in a more toxic effect than pristine CNTs on human T cells due to differences in aqueous dispersibility^31^,^54^.

The *in vitro* results suggested that a possible toxic effect may be encountered after systemic exposure to SWCNTs. Contrary to *in vitro* and *ex vivo* studies, as shown in Fig. 8 and Supplementary Figure S3, none of our compounds resulted in an activation of the immune system *in vivo*, with only the positive control showing altered values of monocytes/macrophages and cytokine production at the first time point (24 h) after the treatment. The lack of *in vivo* activation could be due to the dilution of the injected CNTs by total blood, yielding lower final blood concentrations than those achieved in *in vitro* studies. It has been previously demonstrated that the activation of the innate immunity (e.g. monocytes/macrophages) represents the first event in the response of the immune system to non-biocompatible CNTs^31^. This would be followed by cytokine secretion by monocytes/macrophages, which may activate the helper L_T_ and induce the differentiation of L_B_ into antibody-secreting cells^55^,^56^. Taking this into account and comparing it to our results, we could confirm that at the dose proposed and through the intravenous route, our three compounds remained biocompatible through the duration of the treatment. These results are in accordance to those previously obtained with different types of MWCNTs and SWCNTs^42^,^52^.

In summary, our study showed that all the conjugates had no significant effect on cell viability or activation of RAW 264.7 cells but conjugates **1** and **3** led to a slight but significant increase in IL-6/TNFα, in a dose-dependent manner (*in vitro* studies). All the conjugates resulted in significant reduction in monocyte/macrophage cell numbers within PBMCs and elevated levels of IL-6/TNFα, in a dose-dependent manner, following *ex vivo* exposure, with only SmCl_3_@SWCNT-mAb influencing PBMC cell viability. Monocyte/macrophage depletion was not observed *in vivo* after tail vein injection of 150 μg of the conjugates per mouse. The lack of inducing immunological responses after i.v. injection in mice is encouraging to warrant carrying out future pre-clinical tumor targeting and therapy studies using the radioactive (Sm-153)-filled and Cetuximab-functionalized SWCNTs (**3**).

## Methods

### SWCNT conjugates

As-received Elicarb^®^ SWCNTs (Thomas Swan & Co. Ltd.) were steam- (4 h, 900 °C) and HCl-treated to remove carbonaceous impurities^57^, graphitic particles and metal particles (catalyst). The steam treatment also shortens the length of the SWCNTs, leading to a sample with a median length of 420 nm^58^. Short SWCNTs are of interest when developing delivery vehicles since they present an enhanced biocompatibility. Next, the short and purified SWCNTs were filled with anhydrous SmCl_3_ by molten phase capillary wetting following a previously reported protocol^29^. Empty amino-functionalized SWCNTs [SWCNT-NH_2_ (**1**)], samarium chloride-filled amino-functionalized SWCNTs [SmCl_3_@SWCNT-NH_2_ (**2**)], and filled SWCNTs functionalized with the targeting antibody Cetuximab [SmCl_3_@SWCNT-mAb (**3**)] were prepared according to reported procedures and thoroughly characterized^29^, as described in Supplementary Information. SmCl_3_@SWCNT-mAb conjugate corresponds to samarium chloride filled single-walled nanotubes with sealed ends that have been functionalized with amino groups first, and subsequently with the monoclonal antibody Cetuximab.

Homogenous stock suspensions of the three types of nanotubes at 1 mg/ml were prepared from the solid material by addition of sterile water. The suspensions were diluted to the desired concentrations for the cellular experiments using the appropriate cell culture media (see below).

### Cell culture

#### RAW 264.7 macrophages

RAW 264.7 murine macrophage cell line was obtained from American Type Culture Collection (ATCC). Cells were cultured in RPMI 1640 supplemented with 10% heat inactivated Fetal Bovine Serum (FBS), 100 U/ml gentamycin, β-mercaptoethanol (50 μM) and HEPES (20 mM) under controlled atmosphere (37 °C, 5% CO_2_). When confluency reached 70–80%, RAW 264.7 cells were detached with SE buffer (PBS containing 2 mM EDTA and 2% FBS), reseeded onto 96 well plates at a density of 10^5 ^cells/well and allowed to adhere overnight (37 °C, 5% CO_2_) prior to SWCNT addition.

#### Human PBMCs

As previously described^59^, leukocyte-rich buffy coats from healthy adult donors were obtained from the French Blood Bank (Etablissement Français du Sang, Strasbourg, France) and PBMCs were collected by Ficoll-Histopaque (Sigma-Aldrich 10771) density gradient centrifugation. The experiments were performed on cells from at least 3 different donors. Directly after isolation, PBMCs were incubated for 6 h (37 °C, 5% CO_2_) in 96 well plates (10^6 ^cells/well) in complete RPMI 1640 medium (i.e. containing 10% heat inactivated FBS, 10 mg/ml gentamycin, and 10 mM HEPES; Lonza) prior to be exposed to the SWCNTs.

### Viability and apoptosis in RAW 264.7 and PBMCs

Cytotoxicity of SWCNTs was evaluated by flow cytometry. For this purpose, RAW 264.7 cells and PBMCs were treated with 1, 10, 25, 50 and 100 μg/ml of SWCNT-NH_2_ (**1**), SmCl_3_@SWCNT-NH_2_ (**2**) or SmCl_3_@SWCNT-Ab (**3**). DMSO (20%) was used as death positive control and lipopolysaccharide (LPS, 1 mg/ml) in combination with interferon-γ (IFN-γ, 1 ng/ml) as a positive control for cytokine production. After 24 h incubation, supernatants were collected for cytokine determination and cells were harvested with SE buffer and stained with both APC-Annexin V (AnnV; BD Pharmingen 550475) and propidium iodide (PI, 0.2 μg/ml; Sigma-Aldrich) in a calcium containing buffer for 30 min in darkness.

In addition, in the case of PBMCs, different subpopulations were differentiated by means of cell surface markers (clusters of differentiation [CD]). Mouse anti-human PE-CD3 (BD 555340), FITC-CD19 (BD 555412) and PerCP-Cy5.5-CD14 (BD 555787) from BD Biosciences (Mountain View, CA, USA) were employed to detect and gate T lymphocytes, B lymphocytes and monocytes/macrophages, respectively.

Early apoptosis is shown by AnnV positive staining; double AnnV and PI stained cells are considered necrotic or late apoptotic, while the absence of staining shows viable cells. The percentage of live (AnnV−/PI−), early apoptotic (AnnV+/PI−) and late apoptotic/necrotic (AnnV+/PI+ and AnnV−/PI+) cells was determined by acquiring at least 50,000 events using a Gallios flow cytometer (Beckman Coulter, Villepinte-France) and analyzing the data with Flowing Software 2.5.1.

### Cell activation experiments in RAW 264.7 cells

Flow cytometry was employed to analyze cellular activation through CD86 expression evaluation in RAW 264.7 macrophages. Briefly, cells were incubated with 1, 10, 25, 50 and 100 μg/ml of SWCNT-NH_2_ (**1**), SmCl_3_@SWCNT-NH_2_ (**2**) or SmCl_3_@SWCNT-mAb (**3**). Twenty-four hours later macrophages were detached with SE buffer, washed and stained for 30 minutes with PE-Rat Anti-Mouse CD86 fluorescent antibody (Clone GL1, BD Pharmingen 553692) and subjected to flow cytometry analysis. LPS (1 mg/ml) combined with IFN-γ (1 ng/ml) was used as positive control. Percentage of CD86+ cells was determined by acquiring at least 25,000 events using a Gallios flow cytometer (Beckman Coulter, Villepinte-France) and analyzing the data with Flowing Software 2.5.1.

### Animals

All experimental methods on animal were approved by the UK Home office (PPL 70/7493) and were carried out in accordance with the UKCCCR Guidelines. Twenty female C57Bl/6 mice aged 5–6 weeks (Charles River Laboratories, UK) were caged in groups of four with free access to food and water. A temperature of 19–22 °C was maintained, with a relative humidity of 45–65%, and a 12 h light/dark cycle. Four mice per group and time point were anaesthetized by isoflurane inhalation and intravenously (i.v.) injected with 150 μg of SWCNT-NH_2_ (**1**), SmCl_3_@SWCNT-NH_2_ (**2**) or SmCl_3_@SWCNT-mAb (**3**) in 150 μl of PBS *via* a single tail vein injection. We used only PBS in the negative control group. An intraperitoneal injection of LPS (3 mg/kg) was administered to the positive control group. Thirteen days post-injection the animals were sacrificed by an intraperitoneal lethal dose of pentobarbital. The weight of the animals was monitored throughout the entire experiment.

### Murine immune cells subpopulation analysis

Samples of 150 μl of blood were collected from the tail vein of the animals at specific time points post-injection (1, 7 and 13 days) and PBMCs were isolated to analyze the immunological impact on them. Briefly, heparinized blood was centrifuged at 1000 × g for 10 min. Serum was separated and kept at −20 °C for further cytokine analyses. Remaining sample was washed with 1 ml PBS and centrifuged at 1500 rpm. Supernatant was then discarded and cells were treated for 30 min in darkness with PE Hamster Anti-Mouse CD3e (BD 553063), FITC Rat Anti-Mouse CD45R/B220 (553087) and PerCP-Cy5.5 Rat Anti-Mouse CD11b (BD 561114) in order to stain T lymphocytes, B lymphocytes and monocytes/macrophages, respectively. Afterwards, samples were centrifuged, supernatant discarded and cells treated with ammonium chloride potassium buffer (ACK) to eliminate red blood cells. After a final centrifugation, supernatant was again discarded and cells were resuspended in PBS before being subjected to flow cytometry analysis. The percentage of cells in each subpopulation was determined by acquiring at least 5,000 events using a FACSCalibur flow cytometer (BD, Franklin Lakes, NJ) and analyzing the data with Flowing Software 2.5.1.

### Cytokine determination

#### RAW 264.7 macrophages and in vivo samples

Secretion of IL-6 and TNFα by RAW 264.7 macrophages or by *in vivo* treated mice was assayed using a double-sandwich ELISA. Polyvinyl microtiter plates were coated with 50 μl/well of Specific Purified Rat Anti-Mouse IL-6 (BD Pharmingen 554400) or Specific Purified Rat Anti-Mouse TNFα (BD Pharmingen 557516) diluted in 0.05 M carbonate pH 9.6 buffer and incubated overnight at 4 °C. After washings with PBS containing 0.05% Tween (PBS-T), a saturation step was performed by adding 100 μl/well of PBS containing 10% FBS for 1 h at 37 °C. Plates were then washed with PBS-T and 50 μl of culture supernatants (or serum in the case of mice samples) were added for 2 h at 37 °C. Plates were then washed with PBS-T and 50 μl/well of secondary Biotin Rat Anti-Mouse IL-6 (BD Pharmingen 554402) or secondary Biotin Rat Anti-Mouse TNFα (BD Pharmingen 557432) were added and incubated for 1 h at room temperature. Then, plates were washed with PBS-T, and 50 μl/well of streptavidin conjugated to horseradish peroxidase (diluted 1/500) were added. The plates were incubated for 30 min at room temperature, and then washed extensively with PBS-T and distilled H_2_O. The enzymatic reaction, revealing the presence of cytokines in the tested supernatants, was visualized by adding 3,3′,5,5′-tetramethylbenzidine in the presence of H_2_O_2_. The resulting absorbance was measured at 450 nm after the reaction was stopped with 1 N HCl. Recombinant mouse IL-6 (BD Pharmingen 554582) or recombinant mouse TNFα (BD Pharmingen 554589) were used as standards.

#### Human PBMCs

Secretion of IL-6 and TNFα by PBMCs was assayed using a double-sandwich ELISA following the manufacturer’s instructions in BD OptEIA™ Human IL-6 ELISA Set 555220 and BD OptEIA™ Human TNF ELISA Set 555212, respectively.

### Statistical analysis

Mean values and standard deviation (S.D.) are plotted in each graph. Statistical analysis was performed with SPSS 19.0 (SPSS^®^, Chicago, IL, USA). Normal distribution of samples was assessed by the Shapiro–Wilk test, and homogeneity of variance by the Levene test. The data from the different SWCNT conjugates were compared with ANOVA followed by Bonferroni’s post-test. Statistically significant differences are considered at p < 0.05.

## Additional Information

**How to cite this article:** Perez Ruiz de Garibay, A. *et al*. Evaluation of the immunological profile of antibody-functionalized metal-filled single-walled carbon nanocapsules for targeted radiotherapy. *Sci. Rep.* **7**, 42605; doi: 10.1038/srep42605 (2017).

**Publisher's note:** Springer Nature remains neutral with regard to jurisdictional claims in published maps and institutional affiliations.

## Supplementary Material

Supplementary Information
