# Supplementary material for: Evaluation of the immunological profile of antibody-functionalized metal-filled single-walled carbon nanocapsules for targeted radiotherapy
Source: Sci Rep. 2017 Feb 15;7:42605. doi: 10.1038/srep42605 (PMC5309841; doi:10.1038/srep42605)
Supplement: Supplementary Information [file srep42605-s1.pdf]

Evaluation of the immunological profile of antibody-functionalized metal-filled single-walled carbon nanocapsules for targeted radiotherapy

Aritz Perez Ruiz de Garibay[a,*], Cinzia Spinato[a], Rebecca Klippstein[b], Maxime Bourgognon[b], Markus Martincic[c], Elzbieta Pach[d], Belén Ballesteros[d], Cécilia Ménard-Moyon[a], Khuloud T. Al-Jamal[b,*], Gerard Tobias[c,*], Alberto Bianco [a,*]

[a]CNRS, Institut de Biologie Moléculaire et Cellulaire, Laboratoire d'Immunopathologie et Chimie Thérapeutique, 67000 Strasbourg, France

[b]Institute of Pharmaceutical Science, Faculty of Life Sciences & Medicine, King's College London, London SE1 9NH, UK

[c]Institut de Ciència de Materials de Barcelona (ICMAB-CSIC), Campus UAB, 08193, Bellaterra, Barcelona, Spain

[d]Catalan Institute of Nanoscience and Nanotechnology (ICN2), CSIC and The Barcelona Institute of Science and Technology, Campus UAB, Bellaterra, 08193 Barcelona, Spain

Supporting Information

1. Synthesis of SWCNT-NH_2 (1)

Materials and Methods

Chemical vapor deposition grown SWCNTs were purified by steam treatment. The chemicals and solvents were obtained from commercial suppliers and used without further purification. The solvents used for synthesis were analytical grade. When anhydrous conditions were required, high quality commercial dry solvents were used. Water was purified using a Millipore filter system MilliQ®. When stated, suspensions were sonicated in a water bath (20 W, 40 kHz). For CNT filtration, PTFE membranes from Millipore were employed. Dialysis of CNT compounds was carried out employing membrane with MWCO 12000-14000 Da, purchased from Spectrum Laboratories, Inc. The UV-Vis analyses were performed on a Varian Cary 5000 spectrophotometer and the Kaiser test was performed according to reported procedures.[1,2] Thermogravimetric analysis (TGA) was performed using about 300 µg of sample on a TGA1 (Mettler Toledo) apparatus from 30 °C to 900 °C with a ramp of 10 °C·min^{-1} under N_2 using a flow rate of 50 ml/min and platinum pans. The estimation of the degree of functionalization by TGA was done by taking weight loss values at 650 °C. Transmission electron microscopy (TEM) was performed on a Hitachi H7500 microscope (Tokyo, Japan) with an accelerating voltage of 80 kV and equipped with an AMT Hamamatsu camera (Tokyo, Japan). To prepare the TEM grids, the CNTs were dispersed in a mixture of deionized water and ethanol (1:1) at a concentration of 50 µg/ml using water bath sonication. Ten microliters were deposited onto a carbon-coated copper TEM grid (Formvar/Carbon 300 Mesh; Cu from Delta Microscopies), which was dried under ambient conditions.

Synthesis of SWCNT-NH_2 (1)

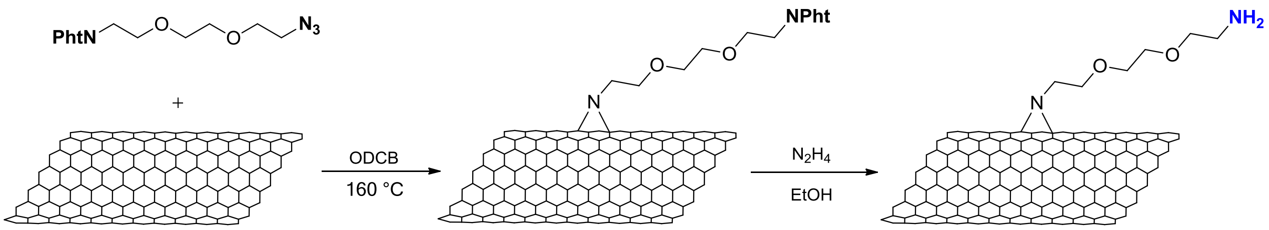

1

In a flame-dried Schlenk tube, pristine SWCNTs (10 mg) were dispersed in dry *o*-dichlorobenzene (ODCB) (8 ml) by sonicating for 15 min under argon. A solution of *N*-[2-(2-azidoethoxy)ethoxy]ethylphthalimide [3,4] (200 mg) in dry ODCB (2 ml), was then added to the CNT dispersion using a syringe, and the mixture reacted for 18 h at 160 °C, under vigorous stirring and under argon. The cooled mixture was then diluted with EtOH (20 ml) and filtered

on a PTFE membrane (0.1 μm). The CNTs recovered on the filter were washed by dispersing them in EtOH (10 ml), sonicating for 10 min and filtrating. This washing sequence was further repeated with MeOH (x2) and with acetone (x2). The CNTs were then dialyzed against distilled H_2O and lyophilized.

For the deprotection, functionalized CNTs (5 mg) were dispersed in EtOH (10 ml) by sonicating for 10 min, and afterwards treated with hydrazine hydrate (0.5 ml). The dispersion was stirred at r.t. for 2 h, and then diluted with EtOH (10 ml) and filtered (0.1 μm). The recovered CNTs were washed with MeOH (x2) and acetone (x2), and finally dried under vacuum. The free amine loading assessed by the Kaiser test is 104 μmol/g.

2. Thermogravimetric characterization of empty SWCNTs (1)

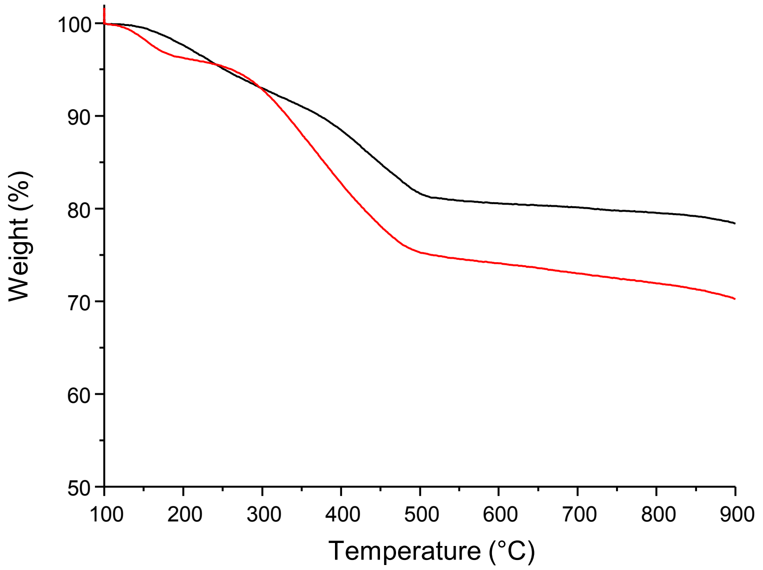

Supplementary Figure S1. Thermogravimetric curves of pristine empty SWCNTs (black) and phthalimide-protected SWCNTs (red).

The Pht-protected precursor of conjugate **1** was characterized by TGA performed under inert atmosphere, confirming the occurrence of the functionalization (weight loss of the phthalimide conjugate is 6.8% at 650 °C, corresponding to a molar loading of 246 μmol/g).

3. Electron microscopy characterization of filled SWCNT conjugates

Electron microscopy proved as a powerful tool to visualize the filling and confirm the successful functionalization of the filled SWCNT conjugates. Low voltage high angle annular dark field (HAADF) scanning transmission electron microscopy (STEM) images were acquired at 20 kV using a FEI Magellan XHR 400L SEM equipped with a dedicated STEM detector. High resolution transmission electron microscopy (HRTEM) images, HAADF-STEM images and energy dispersive X-ray spectroscopy data were acquired at 200 kV on a FEI Tecnai G2 F20 HR(S)TEM equipped with an EDAX super ultra-thin window (SUTW) X-ray detector.

4. Apoptosis in RAW macrophages

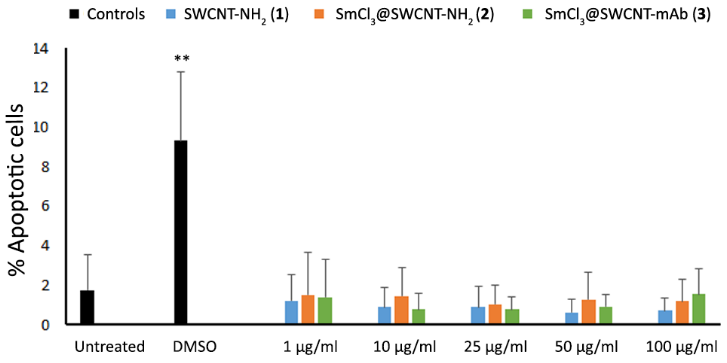

Supplementary Figure S2. Percentage of apoptotic RAW 264.7 macrophages. Cells were incubated with the three SWCNT conjugates for 24 h at increasing concentrations (1, 10, 25, 50 and 100 µg/ml). DMSO (20%) was used as a positive control of death. Cell viability was quantified by flow cytometry and no significant differences were observed for all compounds after 24 h of incubation (n = 3). Values are expressed as mean ± SD. ** $p < 0.01$ with respect to untreated cells.

5. Cytokine production after *in vivo* administration

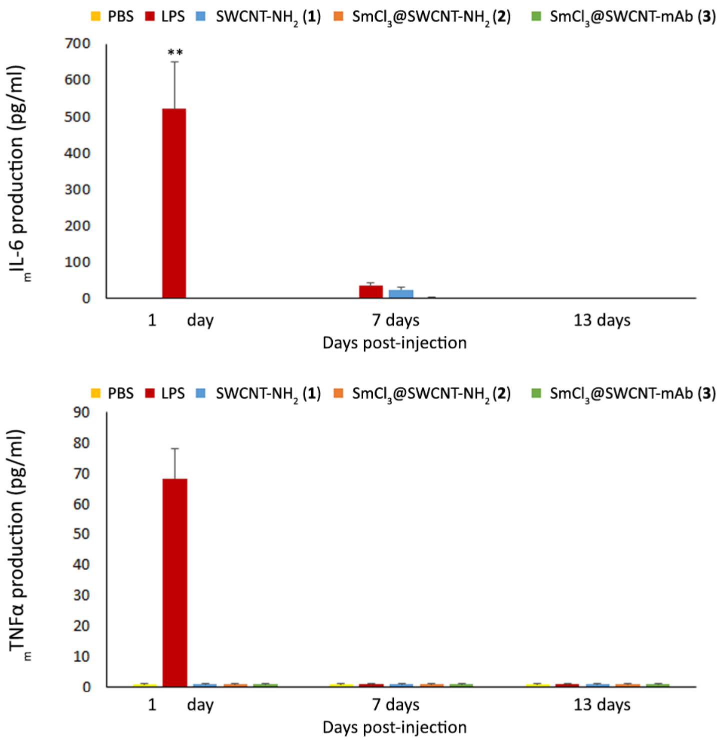

Supplementary Figure S3. IL-6 (top) and TNFα (bottom) cytokine production in mice serum after 1, 7 and 13 days post-injection (150 µl of PBS -negative control-, 3 mg/kg of LPS -positive control- or 150 µg in 150 µl of SWCNT-NH₂, $SmCl_3$@SWCNT-NH_2 or $SmCl_3$@SWCNT-mAb) (n = 4). The absence of bars indicate negligible levels. ** $p < 0.01$ with respect to the other samples.

6. Dispersibility

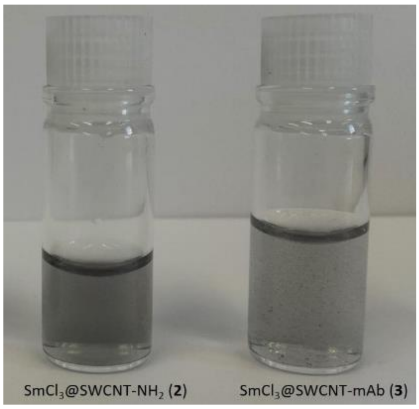

Supplementary Figure S4. Dispersibility of $SmCl_3$@SWCNT-NH_2 (**2**) or $SmCl_3$@SWCNT-mAb (**3**) in PBS (100 µg/ml). Picture was taken 1 min after sonication.

References

1. Kaiser, E. Colescott, R. L. Bossinger, C. D. & Cook, P. I. Color test for detection of free terminal amino groups in the solid-phase synthesis of peptides, *Analytical biochemistry* **34,** 595–598 (1970).
2. Samorì, C. *et al.* Potentiometric titration as a straightforward method to assess the number of functional groups on shortened carbon nanotubes, *Carbon* **48,** 2447–2454 (2010).
3. Spinato, C. *et al.* Design of antibody-functionalized carbon nanotubes filled with radioactivable metals towards a targeted anticancer therapy, *Nanoscale* **8,** 12626–12638 (2016).
4. Lu, G. Lam, S. & Burgess, K. An iterative route to "decorated" ethylene glycol-based linkers, *Chemical communications (Cambridge, England),* 1652–1654 (2006).
